# Inflammatory Status in Trained and Untrained Mice at Different Pollution Levels

**DOI:** 10.3390/ijerph21070821

**Published:** 2024-06-23

**Authors:** Roberta Foster, Mariana Matera Veras, Andre Luis Lacerda Bachi, Jonatas Bussador do Amaral, Victor Yuji Yariwake, Dunia Waked, Ana Clara Bastos Rodrigues, Marilia Farrajota, Robério Pereira Pires, Karina Pantaleão, Juliana de Melo Batista dos Santos, Francys Helen Damian, Paulo Hilário Saldiva, Mauro Walter Vaisberg

**Affiliations:** 1 Department of Otorhinolaryngology and Head and Neck Surgery, Escola Paulista de Medicina, Federal University of São Paulo, São Paulo 04021-001, Brazil; foster.roberta51@gmail.com (R.F.); amaraljb@gmail.com (J.B.d.A.); mariliarf@hotmail.com (M.F.); roberiopereirapires@gmail.com (R.P.P.); kapanta@hotmail.com (K.P.); vaisberg.mauro@gmail.com (M.W.V.); 2 Experimental Atmospheric Pollution Laboratory, School of Medicine, University of São Paulo, São Paulo 01246-903, Brazil; verasine@usp.br (M.M.V.); victoryuji.13@gmail.com (V.Y.Y.); duniawaked@usp.br (D.W.); anaclara.rodrigues@hotmail.com (A.C.B.R.);; 3 Post-Graduation Program in Health Science, University of Santo Amaro, São Paulo 04743-030, Brazil; 4 Department of Physical Therapy, School of Medicine, University of São Paulo, São Paulo 01246-903, Brazil; juliana-mbs@hotmail.com

**Keywords:** atmospheric pollution, physical exercise, inflammation, adaptation

## Abstract

Atmospheric pollution can be defined as a set of changes that occur in the composition of the air, making it unsuitable and/or harmful and thereby generating adverse effects on human health. The regular practice of physical exercise (PE) is associated with the preservation and/or improvement of health; however, it can be influenced by neuroimmunoendocrine mechanisms and external factors such as air pollution, highlighting the need for studies involving the practice of PE in polluted environments. Herein, 24 male C57BL/6 mice were evaluated, distributed into four groups (exposed to a high concentration of pollutants/sedentary, exposed to a high concentration of pollutants/exercised, exposed to ambient air/sedentary, and exposed to ambient air/exercised). The exposure to pollutants occurred in the environmental particle concentrator (CPA) and the physical training was performed on a treadmill specially designed for use within the CPA. Pro- and anti-inflammatory markers in blood and bronchoalveolar lavage (BALF), BALF cellularity, and lung tissue were evaluated. Although the active group exposed to a high concentration of pollution showed a greater inflammatory response, both the correlation analysis and the ratio between pro- and anti-inflammatory cytokines demonstrated that the exercised group presented greater anti-inflammatory activity, suggesting a protective/adaptative effect of exercise when carried out in a polluted environment.

## 1. Introduction

Millions of people around the world are chronically exposed to airborne pollutants at concentrations that are well above recommended standards. The World Health Organization (WHO) estimates that pollution ranks eighth among the main risk factors for mortality [1] and that millions of deaths occur annually as a direct effect of air pollution. Furthermore, a significant number of pathologies are associated with the inhalation of polluted air, affecting practically all organic systems, especially the respiratory and the cardiovascular systems [2].

Pollutants can be classified in several ways, such as primary or secondary. Primary pollutants are those formed directly at the emission source, while secondary pollutants are those formed in the atmosphere due to photochemical reactions. Pollutants are also classified according to the nature of the element in suspension, such as gasses or particulate matter. Particulate matter (PM) is the term used for a mixture of solid particles and liquid droplets found in the air that are small enough to be inhaled [3], categorized by particle size, measured in micrometers (μm), and divided into three types according to their diameter: PM0.1, PM2.5, and PM10.0. The concentrations in the environment are generally quantified as μg/m^3^.

When inhaled, larger diameter PM (>PM10) is limited to the upper airways, while smaller particles (<PM2.5) can access the alveoli. In fact, PM2.5 can also cross the respiratory endothelium to enter the capillaries and, thus, circulate through the blood [4]. PM is also able to carry biological material, such as viruses and bacteria, as well as chemical substances [5].

A study carried out in the USA showed that the lungs are the main target organ for the harmful effects induced by PM, and that they can be characterized according to the effects of exposure to pollutants. In that study, each 10 μg/m^3^ increase in environmental PM10 was associated with a 0.58% increase in respiratory mortality, while the same increase in terms of PM2.5 was associated with a 2.07% increase in hospitalization for respiratory diseases [6].

A study carried out in 187 counties in the USA between 2000 and 2005 identified 52 species of PM2.5, which constituted 83% of the particulate matter, demonstrating that the study of PM basically refers to PM2.5 [7].

The benefits of exercising outdoors in environments with varying degrees of pollution have been discussed for some time.

Physical exercise is an important tool in both the prevention and treatment of a huge range of pathologies, including cardiovascular, metabolic, respiratory, and osteoarticular diseases, among others, and its anti-inflammatory action is one of the main mechanisms of the beneficial effects of exercise [8]. However, it is a fact that most physical exercises (walking, running, cycling, swimming, and even resistance exercises) are performed outdoors, imposing exposure to environmental pollution.

Both atmospheric pollution resulting from traffic and physical exercise carried out in ambient air have increased. This raises a question, as although the literature reports the benefits of PE for the cardiovascular system, doubts persist regarding its effects on the respiratory system, mainly due to the complexity of exposure to multiple pollutants and the varying degrees of exposure [9].

Considering that pollution is associated with an increase in systemic inflammation, how does exercising in environments with different degrees of pollution improve the previously mentioned health conditions?

Several studies registered in the medical literature, involving both humans [10] and experimental models [11], show that exercise is beneficial, even when carried out in a polluted environment. In addition, the literature demonstrates the dangers and benefits of exercising in cities with high levels of pollution. Thus, the current work sought to use an experimental model to copy the pollution from exposure to traffic, focusing mainly on PM 2.5, using the CPA to aid understanding of the harm caused by and the adaptation mechanisms allowing the practice of moderate exercise in ambient air.

## 2. Materials and Methods

### 2.1. Model and Animals for the Study

This is a cross-sectional experimental study, comparing trained and untrained groups of mice that were exposed to different levels of pollutants. It was developed at the National Institute for Integrated Environmental Risk Analysis (INAIRA), at the School of Medicine of the University of São Paulo (FMUSP).

In total, 24 male C57BL/6 (inbred) mice from the FMUSP bioterium were used. The mice were kept from weaning (21 days) until the end of the practical part of the study in the bioterium of the Experimental Atmospheric Pollution Laboratory of the FMUSP Department of Pathology. The bioterium has a HEPA system (High Efficiency Particulate Arrestance) for air filtration, which promotes high retention of very small particles (up to 0.3 microns in diameter).

### 2.2. Animal Exposure and Pollutant Characterization

To evaluate the effects of exposure to particulate matter (PM), experimental animals were exposed to particulate pollutants (PM2.5) with the aid of an environmental particle concentrator (CPA), located at FMUSP. The CPA is a device developed by the Harvard School of Public Health, USA, that uses virtual impactor technology, with the purpose of concentrating the environmental particles present in the atmosphere by up to 20 times without modifying them chemically or physically, allowing control of the concentration to which the animals are exposed and, thus, enabling dose response studies [12].

In the intervals between exposures, the animals were kept in cages on ventilated shelves receiving filtered air, with food and water available ad libitum, and were subjected to a 12/12-h light/dark cycle.

### 2.3. Study Design and Pollutant Exposure Protocol

After the 24 animals reached adulthood (60 days), they performed an effort test (ET) using a Digital Treadmill (Bonther Products and Equipment for Labs Ltd., Ribeirão Preto, Brazil) specially designed for this study protocol to operate within the CPA. The ET was used to define the 4 groups evaluated and to determine the average training speed that the animals reached during the training.

During the effort test, the distance, time, and speed reached by each animal were measured to determine the average speed. Then, 4 groups were defined, taking into account each mouse’s performance during the test: polluted active (AP, *n* = 6)—animals that trained on the treadmill, exposed to a higher concentration of pollutants; polluted sedentary (SP, *n* = 6)—animals that did not train, exposed to a higher concentration of pollutants; clean active (AL, *n* = 6)—animals that trained on the treadmill, exposed to ambient air; and clean sedentary (SL, *n* = 6)—animals that did not train, exposed to ambient air.

The concentrations to which the animals were exposed were determined based on environmental exposures to PM2.5. To maintain a consistent concentration of pollutants throughout the study, the exposure time during which the animals were kept in the concentrator was determined daily. For this calculation, information was collected on the average values of PM2.5 concentration and relative air humidity over the previous 24 h from the CETESB stations in Parque Dom Pedro, in São Paulo, capital. These values were used in a formula to determine the daily exposure time:

(CA/CC) × 60(minutes)

CA = animals exposed = 600 µg·m³ (fixed value)
CC = concentration value inside CPA (value of the day × factor concentration)
Concentration factor = 20 (fixed value).

In this way, the concentration of 600 µg·m^−^³ represents the total average daily concentration to which we are subject in the city of São Paulo.

**Figure 1:**
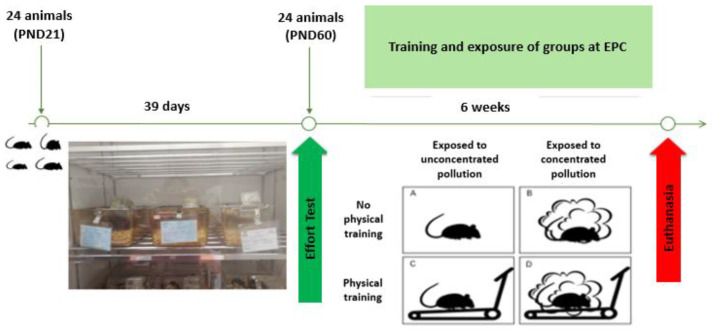
Illustrative diagram of the study design. Photo: personal collection.

It is important to highlight that the animals in the SP and AP groups were exposed to concentrated pollutants for 5 days a week for two hours in the morning, and the animals in the SL and AL groups were exposed to ambient air for 5 days a week for two hours in the morning (Figure 1).

### 2.4. Effort Test and Training Protocol

Before the ET and the 6-week training, the animals were placed in contact with the treadmill for adaptation. For three consecutive days, the mice remained on the switched-off treadmill for about 10 min to walk around the stalls and smell the area. Subsequently, also for three consecutive days, the animals walked on the treadmill at a speed of 0.3 km/h (or 5 m/min), with the testing time changing each day (5, 7, and 10 min, respectively). Finally, also for three consecutive days, the animals walked on the treadmill with changes in speed, maintaining the same total time (10 min): (0.3 km/h or 5 m/min; 0.5 km/h or 8 m/min; 0.7 km/h or 12 m/min, respectively). After 48 h of rest, the ET was performed.

The ET consisted of an initial 3 min when the animals were on the stopped treadmill—for them to adapt—followed by the movement starting, with an initial speed of 0.3 km/h and increases of 0.2 km/h every 3 min until the animal’s exhaustion was reached, verified by the loss of mechanical walking and the animal’s non-reaction (stopped running) after 5 consecutive shocks. The ET training prescription was defined based on an exercise intensity of between 50% (low intensity) and 60% (moderate intensity) as the aerobic threshold.

The training conducted over the subsequent 6 weeks occurred as follows: a warm-up period (beginning of training) and a cool-down period (end of training), lasting 5 min each, and a progressive workload during the 6 weeks. Initially, the training lasted a total of 30 min and every two days 5 min were added, reaching a total time of 60 min in the middle of the third week. The initial training intensity was 20 min (15 min at 50% of TE and 5 min at 60% of TE), for the animals to adapt. Training time at 60% intensity was progressively increased (also every two days), while training time at 50% intensity decreased. Thus, at the beginning of the fourth week the animals maintained 60% of TE throughout the training period [11].

All animals were weighed before and after the familiarization, adaptation, and exercise test (ET) phases, and throughout the training period. Twenty-four hours after the end of training, the animals were euthanized.

### 2.5. Euthanasia of Animals and Collection of Materials

The animals were euthanized by inhaling isoflurane, as recommended by CONCEA (the National Council for the Control of Animal Experimentation) and the Arouca Law (11,794, 10/08/2008), ensuring minimum physical and psychological suffering to the animals, following the recommendations from the American Veterinary Medical Association (AVMA, 2013). Blood and bronchoalveolar lavage fluid (BALF) were collected and the lungs were removed for histopathological evaluation.

The vena cava was accessed to collect blood, and the volume collected with anticoagulant was centrifuged at 1200 rpm at 4 °C for 7 min. The plasma was aliquoted into Eppendorf tubes and frozen in a freezer at −80 °C for subsequent evaluation of the pro- and anti-inflammatory cytokines (IL-1β, IL-10, IL-17, TNF-α, IFN-γ, IL-6, IL-4, and IL-2) by the flow cytometry method. The CBA (Cytometric Bead Array—BD Biosciences Inc., San Diego, CA, USA) flow cytometry kit was used according to the manufacturer’s instructions.

The BALF was collected after the blood withdrawal. A tracheostomy was made in the uppermost part of the trachea (with a special needle attached to a flexible cannula). After this procedure, approximately 1.5 mL of saline solution was injected into the left lung and the bronchoalveolar lavage was performed. This process was carried out very carefully, 3 times, with 500 microliters of solution each time, without removing the cannula. The wash was centrifuged at 1200 rpm at 4 °C for 7 min, and the supernatant was aliquoted into Eppendorf tubes and stored in a freezer at −80 °C for evaluation of the previously described cytokines, also by flow cytometry.

### 2.6. Determination of the Counting and Differential Reading of BALF Cells

After centrifugation and removal of the supernatant, the counting of viable cells and preparation of slides for the differential reading of BALF cells were carried out using the rest of the material.

To make the slides, a volume of 100 µL of the bud suspension formed after centrifugation and removal of the supernatant was used. This volume was centrifuged again on glass slides using a cytology centrifuge or cytocentrifuge (CytoSpin™ 4, Thermo Scientific, Waltham, MA, USA). Differential counting was carried out according to morphological criteria, with 300 cells being counted per slide, and the presence of lymphocytes, monocytes, neutrophils, eosinophils, and epithelial cells was evaluated.

To count the viable cells, after removing 100 µL to prepare the slides, the button at the bottom of the tube was resuspended in 100 µL of HBSS medium (Hank’s balanced solution, +100 µL of bovine serum albumin—BSA 0.1%), and from this volume, the total number of cells were counted in a Neubauer Chamber (model K5-0111, OLEN, Pinhais, Brazil). For this counting process, 10 µL of trypan blue, plus 10 µL of the cell solution, was used. The counting was carried out under an optical microscope (Carl Zeiss, Jena, Germany) with a 40X magnification objective to check cell viability, considering cells stained in blue as dead. This procedure was carried out immediately after collecting and centrifuging the BALF, thus ensuring the characteristics of the cells present in the fresh material.

### 2.7. Determination of Cytokine Concentration in Blood and BALF

Cytokine concentrations were determined in blood and BALF samples using the CBA flow cytometry kit (Cytometric Bead Array—BD Biosciences Inc., San Diego, CA, USA) following the manufacturer’s instructions. To normalize the values obtained from the BALF, the total protein concentration was measured (BCA kit from Pierce™-Pierce Biotechnology, Rockford, IL, USA), also following the manufacturer’s instructions. Corrected BALF cytokine values (pg/µg) were obtained by dividing cytokine values (pg/mL) by the total protein values (µg/mL).

### 2.8. Tissue Collection and Evaluation

The lungs were collected and fixed in 4% paraformaldehyde in PBS (saline) for histopathological evaluation. The preparation of paraffin blocks was carried out in the HistoCore Arcadia C Leica Inclusion Center. Approximately 2 to 3 sections were collected, with a thickness of 5 µm each, that were obtained with the aid of a microtome (Leica RM2125 RTS, Nussloch, Germany) and adhered to glass slides. For histopathological evaluation, staining with Hematoxylin–Eosin (HE) was performed according to the manufacturer’s instructions. The slides were read under an Optical Microscope (Nexcope NE 300—FN 20, Ningbo, China) at 400× magnification.

### 2.9. Statistical Analysis

The statistical analysis was performed using GraphPad Prism software, version 8.1.2. First, the data normality and homogeneity of variance were assessed by the Shapiro–Wilk statistical test and the Levene test, respectively. Parametric data (weight and number of leukocytes in the lung tissue) are presented as medians with standard deviation (X_SD), and the significant differences between the groups were evaluated using Student’s t-test or two-way analysis of variance (ANOVA) with Tukey’s post hoc test for multiple comparisons. Non-parametric data (cytokines values and neutrophil counting in BALF) are presented as median and interquartile range (X_IR), and the significant differences between the groups were evaluated using the Mann–Whitney test or Kruskal–Wallis test with Dunn’s post hoc test for multiple comparisons. In addition, the coefficient correlation analysis, particularly concerning the cytokine values, was performed using the Spearman test (for non-parametric data). Additionally, effect size (ES) was calculated using Cohen’s coefficient, and values between 0.2 and 0.49 indicated a small effect, whilst those between 0.5 and 0.79 indicated a moderate effect, and values higher than 0.8 indicated a large effect. The null hypothesis was rejected at the probability of *p* < 0.05.

The sample size was calculated using G*Power software version 3.1, where the sample size and statistical power were estimated based on the ANOVA test with an effect size of 0.30, at an α level of 0.05 and a statistical power of 0.95. In addition, we based our method on one used in an article with a similar study design [13]. Thus, a total of 24 mice were necessary to perform the present study, which were separated equally per group (*n* = 6).

## 3. Results

### 3.1. Assessment of Atmospheric Conditions and Pollutants

The animals used for the study were exposed to concentrations of PM2.5 in the CPA during a period of 6 weeks, 5 times a week, for a maximum exposure time to the pollutant of 120 min per day. During the remaining periods, they were kept in a bioterium with “pure” air (HEPA filtration system).

**Figure 2:**
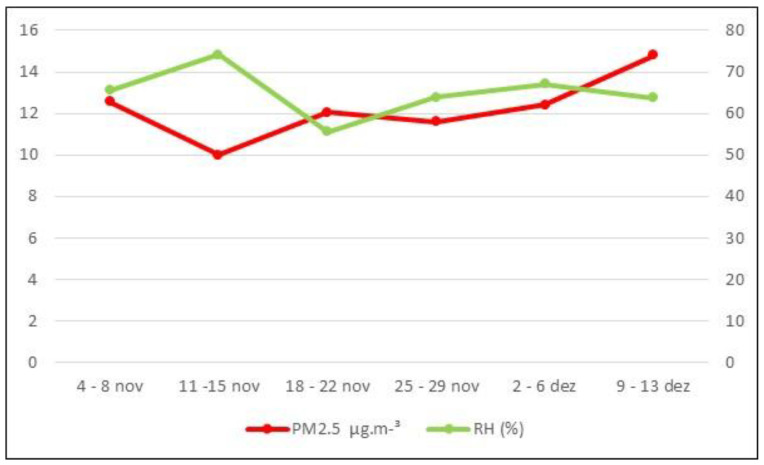
Weekly averages of PM2.5 (µg·m^−^³) and Relative Humidity (%).

**Figure 3:**
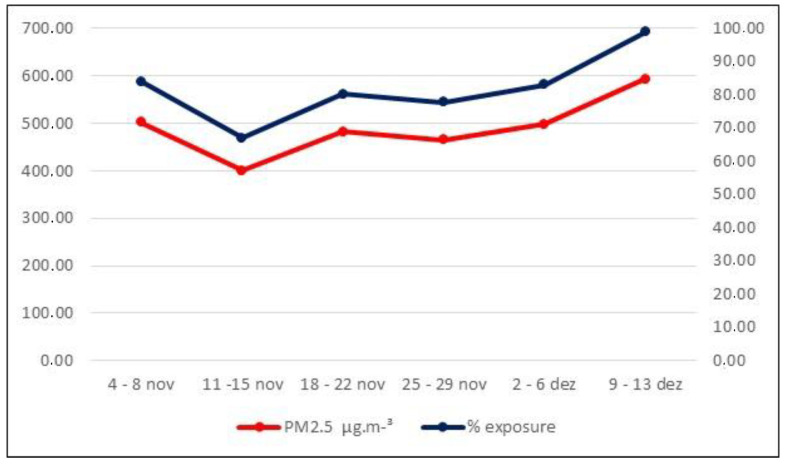
Maximum values (weekly averages) of PM2.5 after exposure (120 min).

Figure 2 shows the weekly averages of PM2.5 and the relative humidity (RH). No weekly RH averages were above 77%, the maximum percentage allowed for exposure to pollutants, which was considered efficient. Figure 3 presents the maximum PM2.5 values inhaled by the animals (weekly averages) after exposure for a maximum time of 120 min (values in µg·m^−^³ and percentage).

### 3.2. Analysis of the Characteristics of the Evaluated Groups

All animals were weighed before and after each training session, throughout the training period. Table 1 presents the averages and standard deviations (SD) of the weight values before and after the study period for all groups evaluated. As expected, in the intragroup analysis using Student’s t-test, higher weights were found at the end of the study compared to the initial values in all mice groups, whereas in the intergroup analysis using the two-way ANOVA with Tukey’s post hoc test, no differences were found between the groups in the study periods.

### 3.3. Analysis of Cytokine Concentrations in Serum

**Figure 4:**
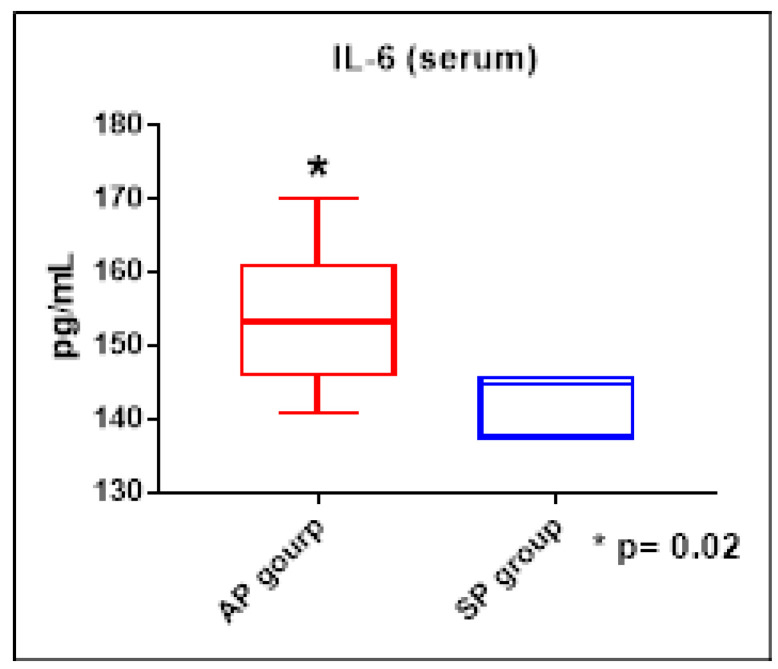
IL-6 serum concentrations (pg/mL) in the AP and SP groups. Mann–Whitney test. * *p* < 0.05.

**Figure 5:**
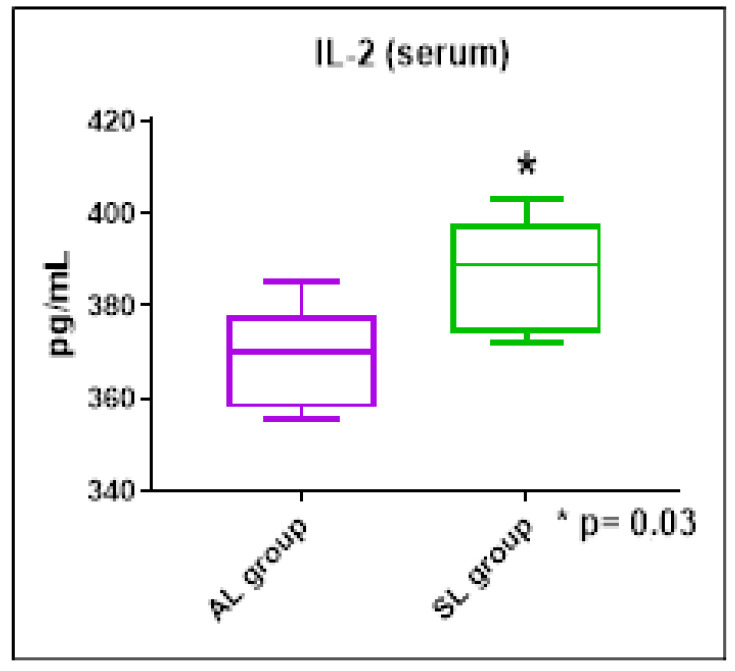
IL-2 serum concentrations (pg/mL) in the SP and AP groups. Mann–Whitney test. * *p* < 0.05.

The results of the systemic concentrations of the cytokines evaluated showed no significant differences between the groups studied. However, we were able to observe higher concentrations of the cytokine IL-6 in the AP group when compared to the SP group (ES = 0.65, Figure 4), as well as increased concentrations of IL-2 in the SL group when compared to the AL group (ES = 0.53, Figure 5).

**Figure 6:**
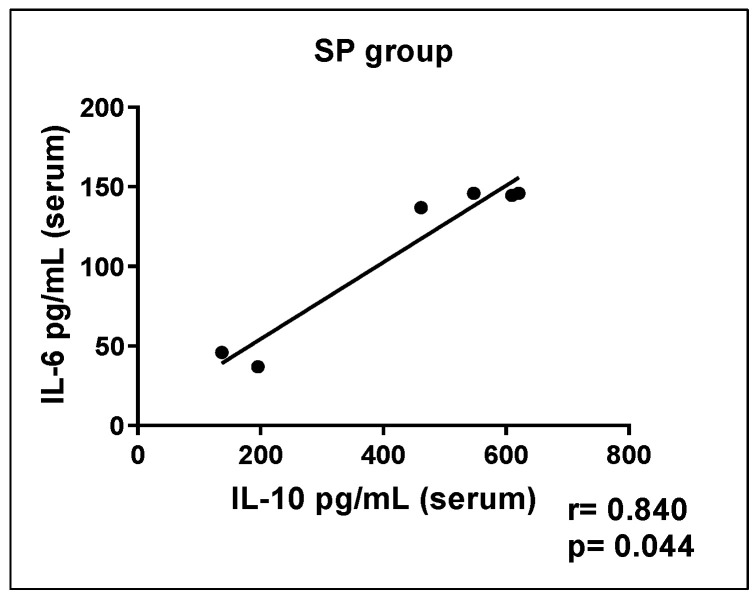
The correlation between serum concentrations of the cytokines IL6 and IL10 (pg/mL) in the SP group.

Correlation tests were carried out among cytokines in the different groups, and the SP group showed a significant positive correlation between IL-10 and IL-6 concentrations (Figure 6). The other groups did not show significant correlation results.

**Figure 7:**
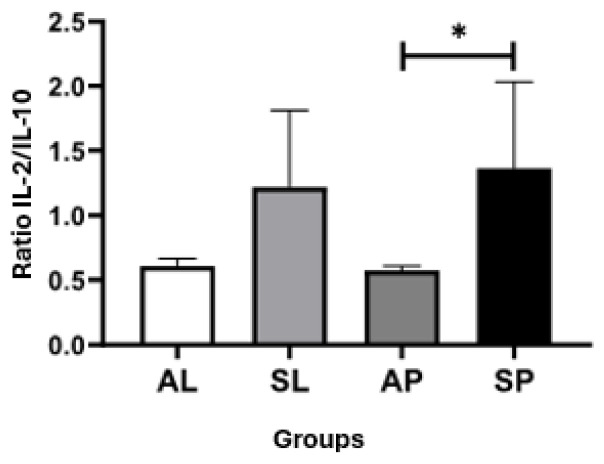
Ratios of serum concentrations of IL-2 and IL10 cytokines between groups. Kruskal–Wallis test with Dunn’s post-test for multiple comparisons. * *p* < 0.05.

Ratio analyses were also carried out between the serum concentrations of the cytokines evaluated in the groups, and a significant difference was observed between the IL2/IL10 cytokine ratios of the SP and AP groups (ES = 0.55), where the SP group showed a higher ratio in relation to the AP group (Figure 7). The other groups did not show significant ratio results.

### 3.4. Analysis of Cytokine Concentration in Bronchoalveolar Lavage (BALF)

**Figure 8:**
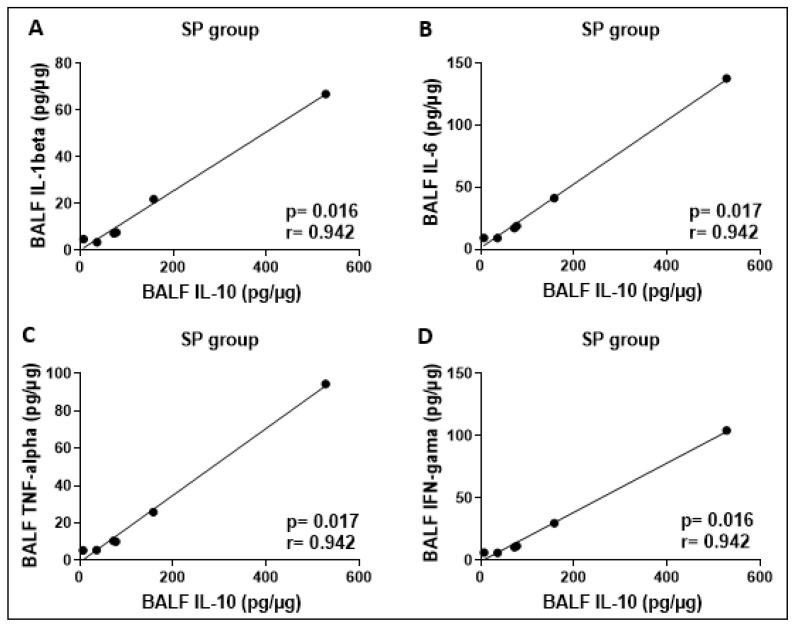
Significant positive correlations between the concentrations (pg/mg) of the cytokines IL-1beta and IL-10 (**A**), IL-6 and IL-10 (**B**), TNF-alpha and IL-10 (**C**), and also IFN-gamma and IL-10 (**D**) were found in the BALF from SP group.

The results of the cytokine concentrations assessed in BALF showed no significant differences between the groups studied. Correlation analyses between cytokine concentrations showed a positive correlation between IL-10 and the cytokines IL-1β, IL-6, TNF-α, and IFN-γ in the SP group (Figure 8). The other groups did not show significant results.

### 3.5. Analysis of Total and Differential Counting of BALF Cells

The total cellularity count in the BALF of the animals evaluated in the different groups was carried out after collecting the material. In all samples the percentage of dead cells did not exceed 2%.

**Figure 9:**
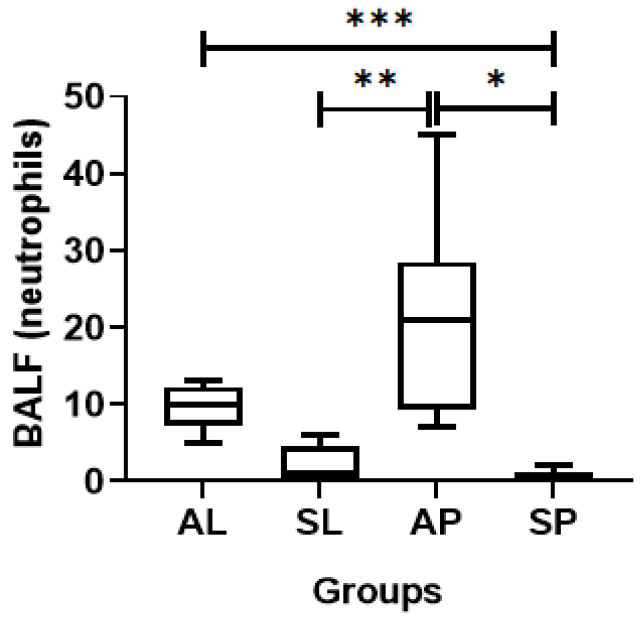
Differential neutrophil counting (absolute number) in the BALF of the different groups evaluated. * *p* = 0.002; ** *p* = 0.02; *** *p* = 0.03.

During the differential counting of BALF cells, no differences in macrophages or lymphocytes were observed between the evaluated groups, but differences were observed in the neutrophils (Figure 9). The AP group presented elevated numbers of neutrophils when compared to the SP group (ES = 0.29) and the SL group (ES = 0.76). In addition, the AL group showed a greater number of neutrophils when compared to the SP group (ES = 1.12).

### 3.6. Histopathological Analysis of Lung Tissue

The histopathological study of lung tissue from the groups of mice exposed to different levels of particulate matter showed an infiltrate of mononuclear cells, with a predominance of lymphocytes and macrophages. Based on this, as shown in Table 2, the mean percentages of lymphocytes and monocytes infiltrating lung tissue were not significantly different between the groups assessed here.

## 4. Discussion

The current study presented some interesting aspects of the airway response of mice exposed to different levels of pollution from particulate matter. The analysis of lung tissue demonstrated an inflammatory response that was similar in groups with greater or lesser exposure to pollutants. However, the study of BALF cytokines did not reflect pulmonary inflammation, suggesting the action of an adaptation mechanism to pollution by the upper airway mucosal immune system.

When we breathe, our body inhales many foreign antigens; however, we do not usually present airway symptoms, because if this were to happen, we would continuously present an inflamed state [14]. However, the harms of pollution are well known, being linked to chronic lung diseases and their worsening; cardiovascular diseases; and metabolic diseases, among others [15].

We observed that mice exercised in an environment with higher levels of pollution showed an increase in the serum cytokine IL-6. It is known that exercise causes an increase in serum IL-6; however, our finding is linked to the inflammation resulting from a higher concentration of pollutants, since the exercising group, at the same intensity but in an environment with a twenty times lower load of pollutants, did not present this increase.

Furthermore, we demonstrated a positive correlation between IL-6 and IL-10 in the group that presented an increase in serum IL-6. This finding suggests an association between inflammatory and anti-inflammatory responses, the first resulting from pollution and the second resulting from exercise, demonstrating that the inflammatory response occurs at the same time as an anti-inflammatory response is being developed.

Another interesting finding is related to IL-2. We observed that the sedentary mice, whether remaining in a clean or polluted environment, had higher serum concentrations of this cytokine than those that were physically trained. According to the medical literature, regular practice of moderate- to high-intensity physical exercise induces a drop in IL-2 levels [16], demonstrating the ability of physical exercise to modulate the systemic inflammatory response.

In addition to these results, another important finding related to IL-2 was associated with the higher value of the IL-2/IL-10 ratio observed in the sedentary mice exposed to pollution when compared to the values obtained in the trained mice exposed to pollution. This result suggests, due to their higher levels of IL-2, an attempt by the sedentary mice exposed to polluted air to increase their production of regulatory T cells (Tregs), to generate a balance in their inflammatory response to pollution, while in the case of the trained mice in a polluted environment, this balance might have already been achieved because physical exercise favors an increase in the regulatory function of Treg cells [17].

Regarding BALF, no differences were observed between the cytokines of the different groups, but we verified a positive correlation between the levels of the anti-inflammatory cytokine IL-10 with the pro-inflammatory cytokines IL-1β, ΤNFα, IFN-γ, and IL-6, particularly in the SP group. This observation is of great value as it demonstrates the attempt to achieve balance in the airways, which leads us to the hypothesis of an adaptation of the mucosal immune system to avoid a state of constant inflammation resulting from continuous contact with material foreign to the organism, as in the case of pollution.

IL-10 is a key cytokine for maintaining an anti-inflammatory state, both in the case of innate and adaptive responses [18].

Among the cell types involved in the oral tolerance process, the most important is probably the Treg cell, and several studies have shown the impact of the type 2 Toll-like receptor (TLR2) on both the direction and function of these cells. The importance of the type 2 Toll-like receptor in the recognition of particulate matter through the airways is clear from the demonstration by Becker et al., (2005) [19]. Horner (2010) confirmed the function of Toll-like receptors in the recognition of non-infectious immunostimulatory materials that stimulate a Th2-type response and observed that when continuous exposure takes place, a tolerance to aeroallergens develops [20]. A study by Yamazaki et al. (2011), shows the induction of IL-10 and Treg cells dependent on Toll-like 2 receptors [21].

Regarding the study of cellularity, we found a statistically significant difference in the number of polymorphonuclear neutrophils (PMN) in the AP group in relation to the SL and SP groups. Wooding et al., (2020), in a study with humans, demonstrated that acute exposure to diesel particles caused an increase in the number of neutrophils in bronchoalveolar lavage [22].

The significant increase in neutrophils in the AP group in relation to the SP and SL groups agrees with several studies of exposure to pollutants in the medical literature that show inflammatory activity in the airway [23].

An unexpected finding was that the number of neutrophils in the lavage was significantly higher in the AL group when compared to the SP group.

In this regard, there is evidence that training in various modalities practiced outdoors in both cold and temperate climates increases the number of neutrophils obtained through the induced sputum technique, therefore presuming an inflammatory condition, but an asymptomatic one, and again raising the hypothesis of an adaptation of the mucosal immune system [24]. It is important to highlight that pollution was not considered in that study.

Regarding the histological study of the lung tissue, we were again surprised by the fact that the histological findings did not demonstrate differences among the groups.

Wang et al. (1992) exposed mice, in cages in a tunnel, to high levels of pollution for between 30 days and 6 months of exposure time. Mice exposed for 30 days showed mainly metabolic alterations, while exposure for 6 months resulted in anthracosis, chronic inflammation, and emphysema [25].

Considering these findings, we can suggest that exposure to PM2.5 is an inflammation factor, even during a short exposure time of six weeks, according to what we were able to confirm in our study. The different PM concentrations did not provoke differences in the histological study among the groups that were exposed to low and high concentrations of pollutants.

Another relevant issue that the study brought to our attention was the fact that the lung histology predominantly showed a mononuclear inflammatory infiltrate, while the cellularity of the bronchoalveolar lavage basically showed polymorphonuclear cells.

In this regard, Winder et al. (1991) studied 70 horses with various inflammatory pathologies and was able to demonstrate a predominance of polymorphonuclear cells in the lavage [26]. Likewise, Naylor et al. (1992), studying horses with chronic obstructive pulmonary disease—an inflammatory pathology—reported that the lavage study revealed a predominance of polymorphonuclear cells [27].

Studies carried out in several animal species have shown that healthy lungs contain a pool of PMN in the alveolar capillaries that is two to three times larger than in peripheral blood, and these cells communicate with other cells of the immune system in this place. The alveoli are overlaid by two types of epithelial cells, and additional cells in the wall of the alveolar septum include endothelial cells, fibroblasts, pericytes, macrophages, and mastocytes. Transmission electron microscopy (EM) studies of the alveolar wall have shown a ‘thin’ side (≤0.2 μm), almost showing an apposition of epithelial and endothelial cells overlaying a shared basement membrane, and a ‘thick’ side, with interstitial components (e.g., extracellular matrix, fibroblasts) separating the epithelium from the endothelium. These findings support the discovery of the PMN predominance in BALF, even in chronic inflammation. These studies propose a model in which PMN migrate during lung inflammation, primarily at the level of alveolar capillaries, suggesting that these cells leave the vasculature in a paracellular manner—in other words, among endothelial cells, in a location where fibroblasts penetrate anatomical holes in the basement membrane, reaching the endothelium and following the trail of these fibroblasts to pre-existing anatomical holes in the subepithelial basement membrane. The paracellular exit of PMNs at the level of the alveolar capillary is generally considered the canonical pathway for PMNs in the inflamed lung, resulting in the predominance of neutrophils in the airspace [28].

It is important to highlight that the literature addressing the topic of pollution provides a large amount of very useful information and raises many questions regarding not only the conflicting results, but also the fact that the methodologies used—such as time of exposure, types of pollutants, and type of exposure—vary widely across studies, making it difficult to provide an overview.

Our study presents some limitations. Although the number of animals was in accordance with the sample calculation, it is possible that a larger number of mice and sequential assessments at different times could help us to more broadly understand both inflammation and the adaptation of the immune system associated with exercise performed in environments with different pollution concentrations.

## 5. Conclusions

In conclusion, our findings suggest that: (a) lung inflammation occurs regardless of the concentration of particulate matter; (b) the upper airway seems to develop adaptation mechanisms to pollution associated with the mucosal immune system, which allows the airway to remain clinically asymptomatic; and (c) physical exercise, despite hyperventilation and greater inhalation of pollutants, is a protective factor against the effects of pollution.

## Figures and Tables

**Table 1:** Averages and standard deviations of weight (in grams) of the animal groups before and after the study period.

Mice (*n* = 24) Groups	Weight (g)		*p*-Value (Effect Size) (Intragroup Analysis)	*p*-Value (Intergroup Analysis)		
AL (*n* = 6)	Before	21.79 ± 0.93	0.0006 (ES = 2.26)		0.1619	
	After	23.63 ± 1.07				
SL (*n* = 6)	Before	21.07 ± 0.91	0.0019 (ES = 1.44)			
	After	24.24 ± 0.54				
AP (*n* = 6)	Before	22.29 ± 0.75	0.0003 (ES = 3.10)			
	After	24.75 ± 0.77				
SP (*n* = 6)	Before	21.44 ± 0.97	0.0110 (ES = 1.29)			
	After	24.04 ± 0.54				

ES = effect size’s value.

**Table 2:** Means and standard deviations (X_±_SD) of the percentages of mononuclear cells, specifically lymphocytes and macrophages, infiltrating lung tissue in the animal groups. Two-way ANOVA with Tukey’s post hoc test.

Mononuclear Cell Type	Mice (*n* = 24)				
	AL (*n* = 6)	SL (*n* = 6)	AP (*n* = 6)	SP (*n* = 6)	*p*-Value
Lymphocytes (%)	78 ± 2	86 ± 5	87 ± 1	83 ± 10	0.2916
Macrophages (%)	13 ± 4	9 ± 4	7 ± 1	8 ± 1	0.1390

## Data Availability

The original contributions presented in the study are included in the article, further inquiries can be directed to the corresponding author.
